# Uncertainties in evaluating the health-related quality of life and disease burden of people with rare diseases and their caregivers in NICE HST submissions

**DOI:** 10.1186/s13023-024-03382-9

**Published:** 2024-10-22

**Authors:** Alissa Looby, Amy Dymond, William Green, Hannah Wentzel, Kinga Malottki

**Affiliations:** 1 York Health Economics Consortium, York, UK; 2 Sanofi, Reading, UK

**Keywords:** Rare diseases, Highly specialised technologies, NICE, Health-related quality of life

## Abstract

**Background and aims:**

The NICE Highly Specialised Technology (HST) programme evaluates interventions for very rare conditions within the UK. This review aimed to analyse previous NICE HST appraisals and determine commonly used methods to overcome uncertainties relating to health-related quality of life (HRQoL) and disease burden for people with rare diseases and their caregivers. The review also aimed to identify areas where further methodological development is required.

**Approach and results:**

A targeted review of all previous NICE HST appraisals published by the 28th February 2022, in which at least one committee meeting had taken place, was conducted. A total of 24 appraisals were included (17 fully completed and seven ongoing). Data were extracted by one reviewer. The evidence review group (ERG) and committee comments were compared against the NICE reference case and synthesised to identify the following methodological uncertainties that occurred most commonly: using alternatives to the EuroQol-5 Dimension (EQ-5D), sourcing HRQoL data from single-arm studies, measuring caregiver disutilities and estimating disease burden.

**Conclusions:**

This review has highlighted the need for new methodology to reflect the impact of the diseases on people with rare diseases and their families. The review identified the following methodological requirements: alternative approaches that should be used when EQ-5D is not appropriate, methods to evaluate paediatric HRQoL and methods to quantify disease burden. This review also highlights the need to establish clear recommendations on the estimation of utilities across different rare diseases.

**Supplementary Information:**

The online version contains supplementary material available at 10.1186/s13023-024-03382-9.

## Background

The Highly Specialised Technology (HST) programme within the National Institute for Health and Care Excellence (NICE) is used to appraise new and existing interventions for rare diseases. Rare diseases are defined as those affecting fewer than five in 10,000 people within the National Health Service (NHS) in England [1, 2]. It is challenging to design and carry out clinical trials for rare diseases within an acceptable time frame and budget, as patient recruitment can be challenging and in addition, these diseases are often poorly understood. More specifically, the natural history of rare diseases is often associated with uncertainty due to heterogeneity and there is a lack of validated endpoints. Small patient numbers also make it difficult to collect robust and meaningful health-related quality of life (HRQoL) data within clinical trials. Therefore, developing new rare disease treatments is often considered challenging [3].

There is, therefore, an unmet need for new treatments for many rare diseases and this has previously led to regulatory incentives that aim to promote relevant research. For example, in 2000, the European Union introduced a series of incentives for designated orphan products, such as a ten-year market exclusivity period and fee reductions during the approval process [4]. In recognition of the challenges associated with developing therapies for rare diseases, and to improve access to these treatments in the UK, NICE has set the cost-effectiveness threshold for technologies reviewed under the HST programme at £100,000 per quality-adjusted life year (QALY) (with an equivalent increase up to £300,000 per QALY for incremental QALY gains greater than 10), as opposed to £20,000 to £30,000 per QALY for technologies reviewed under the Technology Appraisal programme [2].

Demonstrating value for money remains challenging for rare disease treatments submitted to the HST programme despite the higher cost-effectiveness threshold. Capturing the full benefits of treatments for rare diseases involves quantifying both the patient and carer burden. The NICE reference case outlines that, where relevant, the HRQoL impact on both people with rare diseases and their caregivers should be considered. Furthermore, when care might have been otherwise provided by the NHS or personal social services (PSS), it might be appropriate to consider the cost of the time spent providing this care. Nevertheless, the availability of suitable data to support such analyses is often limited, at best.

The generic EuroQol-5 Dimension (EQ-5D) tool is the HRQoL measure for adults that is preferred by NICE [2]. However, this poses a challenge in HST because, while generic HRQoL instruments allow comparisons across diseases, the domains may not necessarily capture the full HRQoL morbidity and mortality impact of rare diseases [5]. For example, the mobility domain of the EQ-5D focuses entirely on walking and, therefore, fails to capture more granular changes in mobility such as the ability of children to crawl or walk without the use of mobility aids [3, 6, 7]. The original NICE guidance (published in 2013) indicated that qualitative empirical evidence on the lack of content validity must be presented to demonstrate that key dimensions of health are missing when the EQ-5D is considered an inappropriate measure of HRQoL [8].

Relevant utility data can be estimated by mapping outputs from other HRQoL instruments to the EQ-5D when such data are not available from either clinical trials or the wider literature. However, this may not be feasible for all rare diseases because disease-specific HRQoL measurement tools are not always available.

Additionally, there are challenges associated with mapping disease-specific measures to the EQ-5D, which can lead to biased estimates if good practices are not followed. Such challenges include: potential misalignments between the concepts being measured, the need for extrapolation outside the sample population, the requirement for appropriate statistical models to be used and adjustments to account for the impact of ageing [9]. A systematic literature review identifying methods to map health state utility values from non-preference-based measures identified 28 studies that used mapping in rare diseases [10]. The authors reported the proportion of rare diseases with a mapping algorithm was very low (less than 2%) compared to the list of conditions screened in the database searches and no mapping studies were found for paediatric rare diseases (likely due to the greater legal and ethical requirements). Challenges of mapping to rare diseases included the requirement to incorporate studies from multiple countries to increase sample size – this leads to inter-country variability in terms of language and culture which can impact estimates. Furthermore, several items included in rare disease-specific outcome measures are unrelated to generic patient-reported outcome measures [10].

Alternative sources of evidence include the identification of data from a systematic literature search, the use of other generic or condition-specific preference-based measures, vignette studies or the use of utility values from a ‘proxy condition’ [2, 11]. The updated NICE manual (2022) supplemented this guidance and acknowledged that it may be problematic to generate HRQoL measures, such as the EQ-5D, for rare diseases [2].

There are also uncertainties associated with estimating the disease burden of rare diseases. The NICE guidelines state that the reference case perspective on costs should be that of the NHS and PSS [2]. Therefore, the productivity costs incurred by both people with rare diseases, and their caregivers, should be excluded from the base case analysis. The guidelines state that analyses incorporating the time spent by family members providing care should be presented separately from the reference case if they are a critical component of a technology’s value proposition [2]. A report published by the NICE task and finish group also states that if costs associated with informal care are collected in trials then they may be included in the economic models (although they are not likely to have a substantial impact on the cost-effectiveness results) [12].

There are two ways in which productivity costs can be quantitively measured: human capital and friction cost. The human-capital approach takes the perspective of the person with the rare disease and/or caregiver and classifies any hours not worked as a loss to the economy. This method assumes that no involuntary unemployment occurs (although this does occur commonly in reality) [13]. The friction cost method takes the employer’s perspective and only considers the hours not worked until the employee is replaced [14].

The objectives of this study were to review previous NICE HST appraisals and determine commonly used methods to overcome uncertainties relating to HRQoL and disease burden for people with rare diseases and their caregivers. In addition, this research aimed to identify areas where further methodological developments are needed.

## Methods

A review of all previous NICE HST appraisals (completed or ongoing) in which at least one committee meeting had taken place (by the 28th February 2022) was completed by one reviewer. Data were extracted from the company submission, the evidence review group (ERG) report and the committee meeting summaries by one reviewer (as detailed in Table 1). ERG and committee comments were mapped to the NICE reference case [2]. ERGs have since been renamed External Assessment Groups, but were referred to as ERGs in this manuscript because the review was conducted on appraisals that were completed before the renaming took place. The review was designed to identify commonly used methods to overcome pre-determined uncertainties relating to HRQoL and disease burden for people with rare diseases and their caregivers. The methodological uncertainties identified from the data extracted were synthesised into the following categories: using alternatives to the EQ-5D, sourcing HRQoL data from single-arm studies, measuring caregiver disutilities and estimating disease burden.

**Table 1:** Elements extracted from HST appraisals

Company submission	ERG report	Committee discussion
• Disease area. • Intervention and comparators. • Economic model perspective. • Description and source of resource use inputs in the economic model. • A qualitative description of the approach to describe the HRQoL and burden on people with rare diseases and their caregivers within the clinical section. • Methods, and input sources, used to estimate the HRQoL of people with rare diseases and their caregivers. • Methods, and input sources, used to estimate the economic burden incurred by people with rare diseases and their caregivers. • Summary of base case results. • Summary of any scenario analyses related to the HRQoL and burden of disease to people with rare diseases and their caregivers.	• Overall consensus from the ERG on submission and economic modelling approach. • Feedback from the ERG regarding the approach to estimating the HRQoL of people with rare diseases and their caregivers. • Any other major criticisms outlined by the ERG relevant to the research question.	• Summary of committee discussions regarding the approach to estimating the HRQoL of people with rare diseases and their caregivers. • The outcome of the appraisal and a summary of how much impact the approach to estimating HRQoL of people with rare diseases and their caregivers had on the overall decision. • A description of the key reasons provided by the committee for recommending/not recommending the treatment for reimbursement.

*HRQoL*,* health-related quality of life, ERG*,* Evidence Review Group.*

## Results

### A summary of HST appraisals

A total of 24 HST appraisals were included in the review − 17 of these appraisals had been fully completed at the time of data extraction (Table 2). A further seven ongoing appraisals were identified in which at least one committee meeting had taken place and the relevant documents were published on the NICE website.

All 17 technologies that had been assessed through the completed process by the 28th February 2022 were approved by NICE in some capacity. A total of ten technologies were recommended and one of these recommendations was optimised (a recommendation that does not cover the full licensed population). Six technologies were approved with a Managed Access Agreement (MAA), in which the intervention was recommended for a limited amount of time while further data were collected to address any clinical uncertainties and inform a future re-appraisal. The MAAs associated with three of these submissions specifically outlined further data collection requirements related to the HRQoL of people with each rare disease and their caregivers [15–17]. For the final submission, the treatment was optimised in one sub-population and recommended via an MAA in a separate sub-population [18].

The number of committee meetings that took place across the HST appraisals ranged between one and five committee meetings (with a median of two meetings per appraisal). A summary of the recommendations, and the associated number of committee meetings, is provided in Table 2.

The range of indications covered in these appraisals displays the wide spectrum of disorders and ages covered by the term ‘rare disease’. Eight of the appraisals were related to lysosomal storage disorders [13, 17, 19–24] and seven were related to metabolic disorders [15, 25–30]. The remaining appraisals covered a range of rare diseases that could not be easily categorised. In terms of age, four appraisals focused on the child population only [16, 18, 27, 31] and five on the adult population only [21, 26, 28, 30, 32]; with the remaining appraisals covering a combination of both.

**Table 2:** Summary of the appraisals included in the review

Reference	Indication	Number of Committee Meetings/ Consultations	Recommendation	Year
*Completed appraisals (at time of review)*				
**HST 1** (33)	Haemolytic uraemic syndrome	Two	MAA	2015
**HST 2** (34)	Mucopolsaccharidosis type Iva	Two	MAA	2015
**HST 3** (16)	Duchenne muscular dystrophy	Two	MAA	2016
**HST 4** (20)	Fabry disease	Two	MAA	2017
**HST 5** (21)	Gaucher disease	Two	Recommended	2017
**HST 6** (15)	Paediatric-onset hypophosphatasia	Five	MAA	2017
**HST 7** (25)	Adenosine deaminase deficiency–severe combined immunodeficiency	Two	Recommended (Optimised)	2018
**HST 8** (27)	X-linked hypophosphatemia	Two	Recommended	2018
**HST 9** (35)	Hereditary transthyretin amyloidosis	Two	Recommended	2019
**HST 10** (36)	Hereditary transthyretin amyloidosis	Three	Recommended	2019
**HST 11** (37)	Inherited retinal dystrophies	One	Recommended	2019
**HST 12** (17)	Neuronal ceroid lipofuscinosis type two	Five	MAA	2019
**HST 13** (28)	Familial chylomicronaemia syndrome	Two	Recommended	2020
**HST 14** (29)	Lipodystrophy	Three	Recommended	2021
**HST 15** (18)	Spinal muscular atrophy	Three	MAA (Optimised) ^†^	2021
**HST 16** (30)	Acute hepatic porphyria	Two	Recommended	2021
**HST 17** (38)	Progressive familial intrahepatic cholestasis	Two	Recommended	2022
*Ongoing appraisals (at time of review)*				
ID 1643** (23)	Mucopolysaccharidosis type IVa	One	NA	TBC
**ID 927** (26)	Erythropoietic protoporphyria	One	NA	TBC
**ID 856** (32)	Emphysema	One	NA	TBC
**ID 1590** (31)	Symptomatic and inoperable plexiform neurofibromas	One	NA	2022
**ID 800** (22)	Alpha-mannosidosis	One	NA	TBC
**ID 737** (13)	Lysosomal acid lipase deficiency	One	NA	TBC
ID 1666*** (24)	Metachromatic leukodystrophy	One	NA	2022

**ID 1643 has since been updated and replaced with HST 19 ***ID 1666 has since been updated and replaced with HST 18.

^†^ *The treatment was optimised in one sub-population and recommended via an MAA in a separate sub-population.*

*MAA: managed access agreement*,* PAS: patient access scheme*

### Sources of utility values

**Fig. 1:**
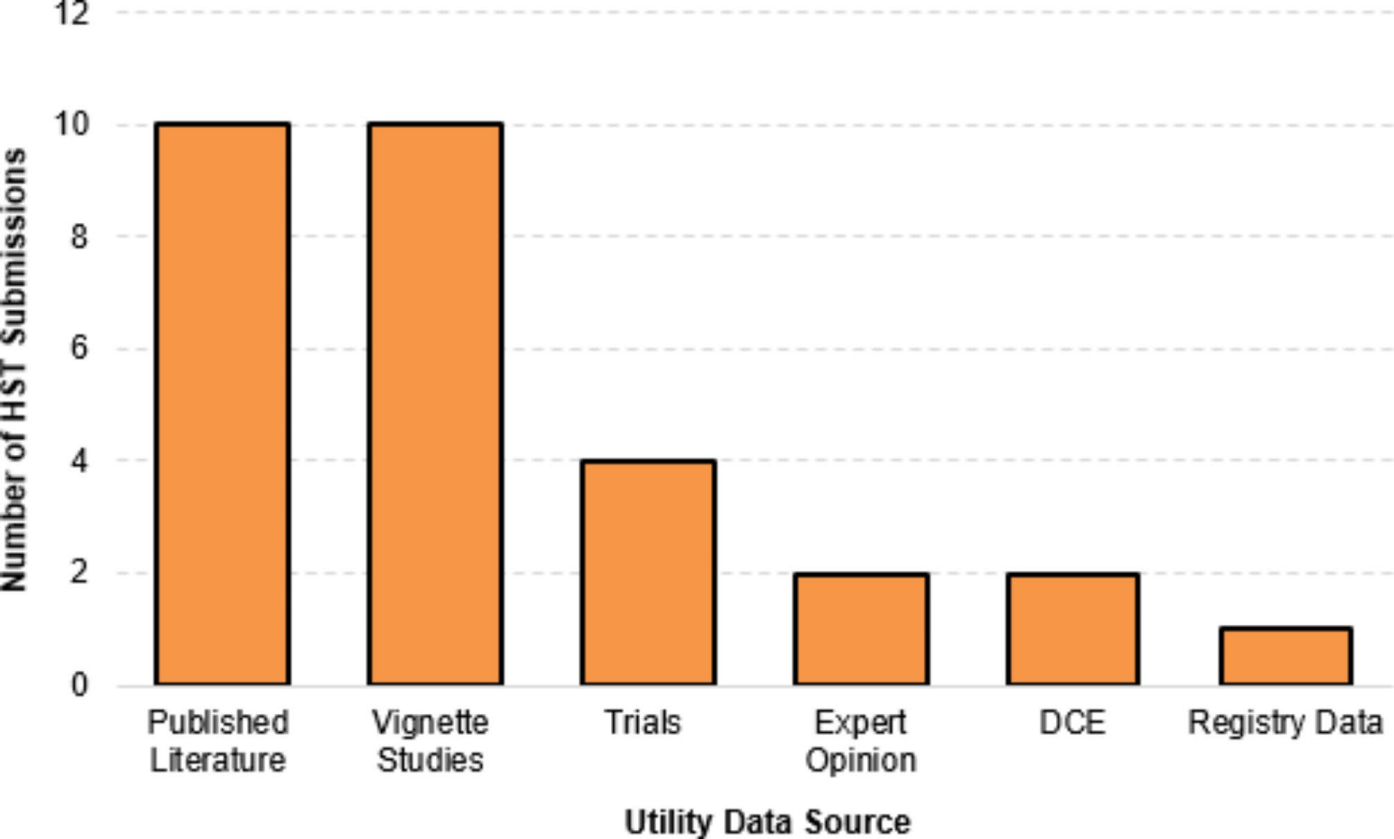
Major sources of utility data used in company submissions. DCE, discrete choice experiment; HST, highly specialised technology

The majority of company submissions used multiple sources to inform utility data that were used in the economic model. Published literature sources were used for the target or proxy conditions in 10 out of 24 HST submissions (Fig. 1). Vignette studies were used to inform utility values within 10 submissions. Other sources, such as submission-specific clinical trials or registry data, were used less frequently. A full list of the sources used to quantitatively estimate utility values in each HST submission is available in Table S1, Additional file 1.

### Alternatives to the EQ-5D

The EQ-5D was used to inform either a proportion of, or all, utility inputs within the economic modelling component of 11 submissions. A wide range of sources were used to identify EQ-5D data – these included published literature sources, mapping other HRQoL tools and EQ-5D-5 L questionnaires being completed by clinicians as a proxy. Justifications for not using the EQ-5D included the domains not providing an adequate perspective on the burden caused by specific disease attributes (including hyperphagia, female reproductive dysfunction, change in physical appearance, and organ abnormality) [29] and counterintuitive EQ-5D results due to small sample sizes and marginal differences in absolute scores [38].

The companies submitting to the HST programme adopted several different methods in the absence of direct EQ-5D evidence. These approaches are described further in the following sub-sections.

The HST submissions that did not inform all, or any, model utility inputs using EQ-5D data contained arguments against the use of the EQ-5D. NICE committees generally accepted that generic HRQoL measures are not suitable for all diseases. However, the use of alternative methods was criticised if not sufficiently justified. For example, the company submitting for HST 13 argued that generic tools were not sensitive enough to detect changes in the HRQoL of people with familial chylomicronaemia (FCS) and, therefore, a treatment benefit could not be captured using the EQ-5D. A clinical expert confirmed that the EQ-5D does not fully capture the detrimental impact of FCS and the committee concluded that the high baseline EQ-5D-5 L values did not reflect testimonies of people with FCS (i.e. this measure was likely to overestimate HRQoL). Furthermore, the committee concluded that intermittent symptoms of FCS are likely to explain why a one-off questionnaire might not capture the effects of the illness on HRQoL [28]. Therefore, the use of alternative methods was considered appropriate for this submission.

#### Paediatric quality of life inventory (PedsQL)

The PedsQL measure was reported in five of the HST submissions reviewed [15–18, 36]. However, HRQoL data obtained using this measure were not commonly used to populate the economic models within these submissions. Whilst discussed qualitatively within the clinical sections, the review did not identify submissions in which alternative paediatric measures were used to inform the utilities of children within the economic models submitted. The predominant reason for this was the challenge associated with mapping the PedsQL to the EQ-5D. The ERGs frequently recommended one algorithm published by Khan et al. [39]. However, this algorithm was based on healthy children and, to the authors’ knowledge, there is currently no validated algorithm to map the PedsQL to the EQ-5D for rare diseases.

#### Vignette studies

Utilities were obtained from specially designed vignette studies in ten of the submissions reviewed [15, 17, 18, 21, 24, 27, 28, 31, 37, 38]. The use of vignette studies in the absence of clinical trial utility data was generally considered acceptable by both the ERGs and committees if appropriate methods were adopted and NICE guidance was followed. However, five submissions were subject to criticism by ERGs and/or committees because the utility elicitation was undertaken by healthcare professionals rather than direct caregivers. One submission (HST 12) was also subject to criticism because the vignettes were separated by treatment arm [17]. Further information on the development and validation of the vignettes used in each HST submission, including a description of the vignettes and the main ERG/committee comments, is provided in Table S2, Additional file 1.

#### Discrete choice experiments (DCE)

DCEs were used to inform HRQoL within two submissions (HST 4 and HST 14); this methodology was criticised in both submissions. The patient disutilities used in the economic model in HST 14 were derived from a DCE completed by the general population [29]. The ERG raised major concerns about the reliability of the methods employed because these were still in early development [29]. The primary concern related to the fact that DCEs classify health states below zero more frequently than time trade-off studies and, hence, produce lower average health state values. As a result, the ERG concluded that the disutilities could only be considered ‘speculative’ until these methodological issues had been resolved. The ERG for HST 4 also noted that it was unclear how comparable estimates from DCEs are to those derived using the EQ-5D and, therefore, the utilities used in the submission lacked face validity.

#### Developing new tools

One HST submission (ID 927) utilised a newly developed disease-specific tool to measure HRQoL (the erythropoietic protoporphyria-specific questionnaire) [26] alongside the Short-Form 36 (SF-36) and Dermatology Life Quality Index (DLQI). The company considered the new tool to be more appropriate in capturing the impact of the disease and treatment than both the SF-36 and DLQI (arguing that most people with erythropoietic protoporphyria reported an unusually high HRQoL at baseline and follow-up). The ERG raised serious concerns about the use of this disease-specific tool and expressed a preference for the DLQI because it has undergone extensive validation, has face validity and has been shown to reflect a marked improvement in quality of life for people with erythropoietic protoporphyria [26]. The ERG did not comment on the appropriateness of using the SF-36 within this submission.

### Sourcing HRQoL data from single-arm studies

It is not always feasible to run randomised controlled trials (the gold standard for efficacy data) for rare diseases due to insufficient trial participants. Furthermore, it may be considered unethical to deny a person active treatment if no other treatment options are available. As such, single-arm studies were used as sources of HRQoL evidence in five company submissions [17, 18, 24, 25, 33]. The company submitting to HST 1 was explicitly criticised for using utility values obtained directly from a single-arm trial [33]. The company subtracted a set value from the utility values in each health state, to estimate the difference in utility following the introduction of the intervention, because the trial lacked a control arm. The ERG stated that the HRQoL data might be subject to confounding and are “at best uncertain” [33]. Alternative sources of HRQoL data were well-received by the ERG and committee when only single-arm trials were available and appropriate justification was provided.

### Caregiver disutility

Fifteen out of the 24 HST submissions (approximately 63%) included caregiver disutilities: eight in the base case and seven in a scenario analysis. The primary reason for excluding caregiver HRQoL from the base case was stated as aligning with the NICE reference case perspective of NHS and PSS [13, 15, 21, 25–27, 33, 40]. Additionally, companies reported that they could not quantify caregiver HRQoL due to a lack of suitable data. A summary of the HSTs that incorporated caregiver disutilities within the submission and, any corresponding ERG/committee comments, is provided in Table S3, Additional file 1.

#### Use of proxy HRQoL data

Ten of the submissions used caregiver disutilities from alternative diseases as a proxy within the economic model [18, 19, 22, 28–30, 35–38]. Only one of these appraisals sought validation for clinical plausibility [22]. A targeted literature search was conducted to identify caregiver disutilities in conditions similar to alpha-mannosidosis. The company identified a paper reporting disutilities for various severity levels of multiple sclerosis - the most appropriate level for each health state was validated by UK-based key opinion leaders.

The use of proxy caregiver disutilities was frequently criticised due to a lack of evidence provided to display that the proxy condition has a similar impact on HRQoL as the condition of interest. The ERG for HST 13 was concerned that the disutilities estimated for caregivers of paediatric patients were applied to caregivers of adults – the ERG believed caregivers of children were likely to incur greater disutility than caregivers of adults [28].

#### Duration for which carer disutilities are applied

12 out of the 15 submissions which quantified carer disutilities applied these disutilities for the duration of the patient’s lifetime [18, 22, 23, 28–33, 35, 36, 41] – this was accepted by the majority of ERGs.

Caregiver disutilities were applied for the whole 95-year time horizon in HST 12 [17]. The ERG argued that the level of caregiver support should be reduced as the person with the rare disease aged. Adults are likely to transition to social care as they age and, hence, the burden for caregivers should be reduced. The ERG suggested a suitable alternative would be to assume that the person with the rare disease would require local residential care following movement into the more severe health states and, hence, this would reduce the caregiver HRQoL burden (i.e. a disutility should no longer be applied). Therefore, the ERG applied the caregiver HRQoL burden for 30 years.

Caregiver disutilities were considered to be only relevant until patients were aged 18 within HST 17 [38]. Furthermore, the company that submitted to HST 11 assumed that the caregiver disutility was halved once patients had left school [37].

#### How many caregivers should be considered?

Eight submissions applied the caregiver disutility to one carer only throughout the model time horizon [22, 28, 30, 33, 37, 38, 41, 42]. The assumption of one caregiver was accepted by the ERGs in all but one submission, in which the ERG suggested a total of 1.78 caregivers should be assumed (equating to the mean number of parents in a household). The company submitting for ID 1590 assumed 1.4 caregivers (one minus the average household size) and the ERG proposed a value of one should be used instead [31]. However, an assumption of 1.67 caregivers used within HST 14 was not queried by the ERG or committee [29].

The number of caregivers differed by disease severity within three submissions [24, 35, 36]. It was assumed that one caregiver would be required whilst the patient was in the initial health states and this value increased to two when the patient had progressed within HST 9 and 10. Similarly, the model for ID1666 assumed that no caregivers would be initially required and two would be required following progression. The company submitting for HST 12 assumed that the number of carers would vary by health state (ranging from 0.06 to 1.14) [17]. Further information on the number of caregivers within the submissions reviewed, and any corresponding ERG/committee comments, are provided in Table S3, Additional file 1.

#### Bereavement

Caregiver disutility associated with bereavement was formally included in the economic model in two of the company submissions reviewed [22, 25].

The company submitting HST 7 included a disutility for bereavement following the death of a child within a scenario analysis which decreased the incremental cost-effectiveness ratio by £3,159 relative to the base case (from £36,360 to £33,201) [25]. Whilst the ERG did not comment on this disutility, the committee did not believe it was appropriate to incorporate it due to the lack of disease-specific data to inform the analysis. Therefore, this scenario was not used to inform the approval decision.

In ID 800, the disutility related to bereavement was included in the base case [22]. The company assumed that patients simulated to not recover from a severe infection would enter a short-term end-stage health state (the most severe health state in the model). The patient was assumed to die within four weeks of entering this health state. However, a caregiver utility decrement was applied for the full year to account for the prolonged bereavement. The ERG did not specifically comment on this approach but presented a range of scenario analyses in which these caregiver disutilities were excluded.

The company submitting for HST 15 quantified the emotional burden on caregiver HRQoL whilst their children were alive, but did not attempt to quantify bereavement following death [18]. The ERG asked the company why the HRQoL impact on bereavement was excluded and the company responded by stating there were numerous methodological challenges in quantifying the emotional impact of losing a child. The company noted that the methods were still in their infancy and did not consider it appropriate to quantitatively estimate the HRQoL impact of bereavement. The ERG agreed with the justification provided by the company.

### Disease burden

#### NICE reference case: disease burden

Whilst all the HST submission dossiers that were reviewed included a narrative description of the burden of disease incurred by people with rare diseases and their caregivers, only 11 of the submissions included a quantitative estimation of burden in either the base case or as a scenario analysis [15–18, 20, 22, 26, 33, 37, 38, 43]. Of these submissions, five submissions included wider societal costs in the base case analysis [15–17, 33, 38]. The most commonly reported reasons for companies excluding these burdens were to ensure the NICE reference case was followed and difficulty in quantifying the true impact of rare diseases. Common topics that were described included:

- The financial and psychological impact of people with rare diseases and caregivers not being able to work or study (this included caregivers often being required to use annual leave for care duties).
- Out-of-pocket costs associated with travelling to health care appointments.
- The psychological impact of people with rare diseases being confined to wheelchair use.
- The emotional impact of people with rare diseases, and their caregivers, not being able to socialise (due to not being well enough, and not having the time, respectively).
- The impact of a rare disease on relationships for both the person with a rare disease, their caregivers and other family members including siblings.
- The burden associated with the requirement to adapt houses and/or vehicles or move home to be closer to a specialist centre.

Two submissions (HST 1 and HST 17) considered wider societal costs in the base case analysis [33, 38]. The ERG provided criticism on the inclusion of such costs in the base case analysis and/or the methods employed to derive these costs in these submissions. Seven of the submissions included societal costs within a scenario analysis [13, 18, 20, 22, 23, 26, 37]. Whilst the ERGs did not criticise companies for reporting these scenario analyses, some of the methods employed to quantify the costs were criticised. The ERG also criticised the company for not quantifying the additional expenses incurred by people with the disease or their caregivers in two of the submissions (HST 14 and ID 737) reviewed [13, 29].

One submission (HST 12) quantified sibling disutility to represent the impact on quality of life felt by siblings unaffected directly by neuronal ceroid lipofuscinosis type 2 (CLN2) [17]. The disutility was obtained from a report on the challenges of living with and caring for a child affected by CLN2 (data on file, provided by the company). The disutility was applied to an average of 0.94 siblings in all but the first two fortnightly health states – the company justified this through clinical expert opinion that the impact on the sibling increases as disease severity worsens over time. The ERG considered the inclusion of sibling disutility to be appropriate given the substantial impact of CLN2 on family life (the committee also agreed with this observation). They also argued that the disutility should have not been applied for the entire lifetime horizon because healthy siblings are likely to leave home. However, overall its removal had minimal impact on the cost-effectiveness results.

#### Human Capital vs. friction cost

The most discussed aspect of the quantification of rare disease burden was the use of the human capital approach vs. the friction cost method. Six of the company submissions reviewed referred to only the human capital approach [15, 16, 19, 33, 38, 41]. The company submitting for ID 737 presented results for both the human capital and friction cost approach [13]. Sensitivity analysis conducted by the ERG illustrated the possible differences depending on the method used to calculate productivity losses. The results showed a productivity loss of £38,096 when using the human capital approach and £2,226 when using the friction cost method [13]. Within the appraisals reviewed, the ERGs concluded the estimated burden associated with rare diseases is likely to be greater when using the human capital approach compared with the friction cost method. Therefore, the friction cost approach was preferred over the human capital approach because it is more conservative.

## Discussion

All HST appraisals examined as part of this review qualitatively described the impact of the disease burden on patient and caregiver HRQoL within the clinical sections of the original submissions. However, considerable uncertainty associated with the quantification of these aspects was found across every submission – this uncertainty was largely attributed to the range of methods used to obtain utility values to inform the economic model. Whilst some methods were critiqued, or considered highly uncertain, the EQ-5D remained the most well-received source of utilities.

This review provides a comprehensive analysis of all HST appraisals that were completed or ongoing (with at least one committee meeting) at the time of data cutoff. An attempt was made to capture all the relevant perspectives: that of the submitting company, the ERG and the committee. However, some relevant information was redacted as confidential because this review was based on publicly available documents and was, therefore, not available for analysis. In addition, it is likely that some of the committee discussions were not completely reflected in the public documents, owing, for example, to their confidential nature. Furthermore, the review does not consider any HST appraisals published after the data extraction was conducted (18th February 2022).

To the knowledge of the authors, one similar study has been published previously. Research published by Pennington et al., provided information on the methods and data sources used to incorporate caregiver HRQoL within previous NICE technology appraisals and HST submissions [44]. The present study provides further insight because it also examines the inclusion of caregiver or patient burden.

The review highlights the difficulty in obtaining suitable utility estimates for use within rare disease submissions. In particular, whilst the NICE reference case requests that the EQ-5D instrument is used, the outcomes of the review reinforce that this instrument is often unsuitable for capturing the HRQoL implications of rare diseases. However, the evidence required to demonstrate the inappropriateness of using the EQ-5D to capture the HRQoL impact of a rare disease is extremely challenging for companies and a more flexible approach is required.

NICE recommends data from elicitation studies (such as vignettes) are used when EQ-5D data are not available or unsuitable for the rare disease [12]. These studies, which are often run specifically to inform a NICE submission, are expensive and time-consuming to conduct. For example, the development of robust vignettes requires extensive qualitative work (e.g. in-depth interviews and focus groups) and the vignettes must be validated by people with rare diseases – these people may be difficult to identify and recruit [45]. The methods used within these elicitation studies are considered to be weak when used for non-rare diseases. Therefore, their widespread use and acceptance within rare diseases highlights this difficulty further.

Whilst vignettes were generally viewed as an acceptable source of utility data, it is clear that these should be completed by people with the disease or caregivers (rather than clinicians) and be appropriately validated to ensure that the health states presented are generalisable to the specific rare disease. There were, however, multiple instances in which methodological limitations of the vignette studies were highlighted. In particular, NICE guidance indicates that, where possible, HRQoL should be measured directly by people with the disease. If people with the disease cannot complete the exercise, these measurements should be completed by direct caregivers rather than clinicians. Contrastingly, the ERGs and committees praised vignette studies that were either developed or validated by clinicians.

Companies were often criticised for the use of DCEs to inform HRQoL within HST submissions due to methodological issues associated with this elicitation method. The ERGs and committees considered DCEs to underestimate utility values and stated that further evidence is required to inform the usefulness of DCEs in rare diseases [45]. Further research and guidance on the use of DCEs is, therefore, required. The use of utility data from a single-arm trial is also likely to be critiqued by the ERG and committee unless there is a justifiable reason to use these trials as an evidence source.

Companies face additional challenges when obtaining HRQoL data for treatments licensed for children and young people. Whilst NICE does not recommend specific measures of HRQoL in children and young people, the PedsQL is one instrument that can be used to provide a general measure of HRQoL in this population and was deemed acceptable by the ERGs and committees [46]. However, even this measure is associated with serious limitations. In particular, it may underestimate the HRQoL of people with severe diseases, in which some very severe health states can never be considered worse than death because it is bound at zero [45]. Furthermore, the Khan algorithm, which was recommended by some ERGs, is not validated across all diseases and may not fully reflect disease severity since it was based on healthy children [39].

Caregiver disutility should only be applied for the period in which there is a direct negative consequence of the disease on the caregiver. While caregiver HRQoL and burden of disease were qualitatively captured in all previous appraisals, the ERG and committee comments indicated that these aspects should also be quantitatively assessed within the economic modelling because they are an important consideration for rare diseases. The recommendations were generally that these aspects should be reported separately from the base case analysis (i.e. as a scenario only).

There is considerable uncertainty associated with the quantification of caregiver utilities within rare diseases – these relate to both the impact and duration of care required. The use of utility data from a proxy condition is often required due to very limited data available on rare diseases – this is likely to be considered acceptable by ERGs and committees if the similarities between the two diseases, in terms of population demographics and clinical equivalence, can be proved. It is also necessary to provide evidence that the proxy disease has a comparable HRQoL and burden to the rare disease for which the new treatment is licensed. Furthermore, the number of caregivers per person should be considered carefully. The results of the review suggest ERGs and the Committee conservatively assume only one caregiver per person unless evidence can be provided to demonstrate that more than one caregiver is affected.

Whilst caregivers often experience disutility due to bereavement following the death of a person with a rare disease, a NICE task and finish group report stated that the effect of bereavement should not be included in economic models because the methods for the inclusion of bereavement are not well developed [12, 47]. Additionally, the impact of bereavement is subjective and difficult to predict. As in several rare diseases, issues of grief and bereavement are of utmost importance and the development of sound methodological guidelines for incorporating this in economic models is required.

The burden on people with rare diseases, and their caregivers, is frequently underestimated. Specifically, people will need to take time off from work and normal social functioning, to conduct activities such as appointment attendance and providing care. The ERGs expressed a preference for the friction cost method over the human capital approach throughout the submissions that quantitatively estimated patient and caregiver burden.

## Conclusion

This review has highlighted the need for new methodologies to reflect the impact of rare diseases on people with the diseases and their caregivers, including new approaches to use when the EQ-5D is not appropriate, new ways to evaluate paediatric HRQoL, further research and methodological advances to quantify bereavement and ways to quantify disease burden. These findings are in line with previous research into the estimation of health-state utility values in rare diseases, which also highlights the need to establish clear recommendations on the estimation of utilities across different rare diseases [45]. These methodological advancements could also help to reduce unavoidable uncertainties associated with rare disease appraisals, which can limit access to rare disease treatments [48].

## Electronic supplementary material

Below is the link to the electronic supplementary material.

Supplementary Material 1

Supplementary Material 2

Supplementary Material 3

Supplementary Material 4

## Data Availability

All data generated or analysed during this study are included in this published article and its supplementary information files.

## Abbreviations

DCE: Discrete choice experiment
DLQI: Dermatology Life Quality Index
EQ-5D: EuroQol-5 Dimension
ERG: Evidence review group
FCS: Familial chylomicronaemia
HRQoL: Health-related quality of life
HST: Highly Specialised Technology
MAA: Managed Access Agreement
