# Supplementary material for: Uncertainties in evaluating the health-related quality of life and disease burden of people with rare diseases and their caregivers in NICE HST submissions
Source: Orphanet J Rare Dis. 2024 Oct 22;19:391. doi: 10.1186/s13023-024-03382-9 (PMC11494764; doi:10.1186/s13023-024-03382-9)
Supplement: Supplementary file 3 — Supplementary Material 3 [file 13023_2024_3382_MOESM3_ESM.docx]

# Supplementary tables

Table S1: Summary of approaches used to quantitatively estimate utility within reviewed HST submissions

| **HST** | **Population** | **Source of Utilities Used in Economic Modelling** | **Key ERG/Committee Comments** |
| --- | --- | --- | --- |
| **HST 1** | Children and adults with aHUS | EQ-5D data collected within clinical studies were used to estimate the weighted improvement in mean utility score from baseline to week 64. | **ERG:** Utilities may have been subject to confounding as they were drawn from single-arm studies which did not include a control group.  **Committee:** Other substantial benefits had not been adequately captured in the model and, therefore, might have led to an underestimation of the cost-effectiveness of the intervention. |
| **HST 2** | People with Mucopolysaccharidosis type IVa | BOI study and clinical opinion.  Additional Delphi exercise, with seven clinical experts, was required because the BOI study did not provide utility value for one health state.  A utility increment was applied to the treatment group. | **ERG:** Questioned the appropriateness of applying a utility increment to the treatment group – likely to result in double counting.  **ERG:** The BOI study reported adult and child utilities but only adult utilities were used in the model with no reason stated (child utilities show a different pattern to adult utilities).  **Committee:** Requested further utility values were collected as part of MAA. |
| **HST 3** | People 50-years and older with DMD | Obtained utility weights for patients and carers HUI and EQ-5D obtained from a study identified in the SLR (47).  Applied the same utility values to both treatment arms. | **ERG:** Explored whether a utility value applied to someone who had just lost their ability to walk should be applied for their lifetime (i.e. would the person would adapt).  **ERG:** Explored whether people receiving the intervention would have a different HRQoL from those on best supportive care.  **Committee:** Requested HRQoL data be collected as part of the MAA. |
| **HST 4** | People with Fabry disease | Literature informed the main utility values used in the model (37).  Infusion-related utility decrements informed from DCE.  AE disutilities were obtained from a published study that used the EQ-5D (48). | **ERG:** Uncertainty around the estimates chosen for the disutility associated with ERT infusion - the disutility was considered unrealistic as it was larger than that associated with stroke or cardiac complications.  **Committee:** migalastat is associated with more health benefits than ERT but the committee preferred ERG estimates to the company estimates. |
| **HST 5** | Adults with type one Gaucher disease | Health states defined using the Gaucher disease type one severity scoring system (GD-DS3).  Used the results of the ‘DS3 Study’ which collected SF-36 and mapped it to EQ-5D.  Utility increment associated with oral administration: Vignette study  Published estimates were used to inform the disutility associated with AE. | **ERG:** The GD-DS3 scoring system is insensitive to changes in disease status and does not reflect differences between the treatments that are observed in the trial.  **ERG:** Uncertainty associated with the utility increment assigned to oral treatment in comparison to infusion.  **Committee:** Understood the utility increment was a key driver of QALY benefits and concluded that even though the true value was uncertain, the alternative value provided by the ERG (0.05 compared to 0.12) was more appropriate. |
| **HST 6** | People with paediatric-onset hypophosphatasia | Vignette study | As described in Table S2 |
| **HST 7** | People with adenosine deaminase deficiency-severe combined immunodeficiency (ADA-SCID) | Utilities derived from external literature (duration informed by clinical opinion).  Utilities defined by long-term survival were derived from the general population EQ-5D scores.  Health states for the six months post-procedure were obtained using vignettes in corresponding studies. | **ERG:** Preferred base case predicts lower QALYs associated with the intervention as a result of increased mortality and morbidity associated with rescue transplants and the application of HRQoL decrements for intravenous immunoglobulin (IVIG) use and bilateral hearing impairment.  **Committee:** Values were highly uncertain but noted that HRQoL gains were similar between the ERG’s preferred approach and that of the company.  **Committee:** Concluded the ERGs assumptions were preferable for decision making as these were based on the available evidence. |
| **HST 8** | Children and young people with X-linked hypophosphataemia | Vignette Study | As described in Table S2 |
| **HST 9** | People with hATTR | Health state utility values were obtained from published literature (49).  **After consultation:** Company used EQ-5D health states which were closest to the mean disease stage values for people in the Transthyretin Amyloidosis Outcome Survey (THAOS) registry. | **ERG**: Utility data used in the model were based on Brazilian valuations which were not likely to represent UK general population preferences for EQ-5D health states.  **Committee:** The utility values used in the model were highly uncertain and although the company’s revised approach was not optimal it was acceptable for decision-making. |
| **HST 10** | People with hATTR | Utilities from company trial (EQ-5D-5L mapped to EQ-5D-3L).  Utilities were capped with a maximum and a minimum using Kind *et al*., 1999 (50).  Utility increased every month the patient received treatment.  Confidential regression analysis was conducted.  **After consultation:** The company ran an analysis where it eliminated the caps and any improvement. | **ERG**: Considered the regression to be unreliable because it excluded important parameters, including the interaction of time by treatment without the main terms and the company chose the maximum and minimum caps arbitrarily.  **ERG:** Also had concerns regarding how the utilities for people who had stopped treatment were modelled and the fact that any improvement in HRQoL was time-dependent.  **Committee:** The committee was satisfied with the assumptions of the company’s scenario analysis (post-consultation). |
| **HST 11** | People with inherited retinal dystrophies caused by RPE65 gene mutations | Vignette study | As described in Table S2 |
| **HST 12** | People with a confirmed diagnosis of neuronal ceroid lipofuscinosis type two | Vignette Study | As described in Table S2 |
| **HST 13** | Adults with confirmed familial chylomicronaemia syndrome | Vignette Study | As described in Table S2 |
| **HST 14** | People with generalised or partial lipodystrophy | DCE used to inform health state utilities.  Utility decrements associated with organ damage were obtained from published literature and based on other conditions. | **ERG:** The DCE methods used to obtain disutilities are still in their infancy.  **Committee:** Agreed with using utility decrements from other conditions given the scarcity of data for this condition.  **Committee:** Expressed concerns relating to the validity of the utilities from the DCE.  **Committee:** Generally satisfied that the company included a utility differential to account for symptoms related to organ damage that was not captured by estimates of utility. |
| **HST 15** | Children with SMA type one | Most health state utilities were obtained from published literature using the EQ-5D-3L (51).  The remaining health state utilities were obtained from previously published US ICER Report and clinical advisors.  The utility increment whilst on treatment was also obtained from the ICER Report (52). | **ERG**: Recognised the difficulties in obtaining accurate utility values and supported the company’s methods.  **Committee:** Considered there to be uncertainty around the health state utilities in the model and that these had a major impact on the cost-effectiveness; however, these methods were considered the most appropriate. |
| **HST 16** | Adults and young people aged 12 years or older with recurrent severe attacks of AHP | The company collected data in the ENVISION study, however, these were not used in the company’s base case.  The company adopted a utility decrement approach for the base case values. Utilities were adjusted for gender and age, and then disutilities associated with acute attacks and long-term chronic symptoms were applied to estimate health state utility values.  The company’s clinical study (EXPLORE) was used to inform the utility values associated with acute attacks. This is a natural history study which assessed EQ-5D-5L at baseline, six months and 12 months. | **ERG:** The base case values were subject to uncertainty as these were derived from published literature. The ERG noted that data was collected in the ENVISION study but these data were not utilised in the economic model. The company stated they did not do so because the values lacked face validity.  **ERG:** Health state utilities should be based on RRMS utilities as reported in Hawton et al (53).  **ERG:** Noted that the use of the EXPLORE study was subject to uncertainty given the differences in key characteristics.  **ERG:** Overall, the ERG agreed with the company that ENVISION data were unlikely suitable for use (given the paucity of attack disutility data).  **Committee:** The committee did not consider the ENVISON utilities plausible because they suggested higher HRQoL in more severe health states.  **Committee:** The company’s approach of summing the effect of single chronic symptoms was flawed. |
| **HST 17** | People with progressive familial intrahepatic cholestasis (PFIC) | Mapped PedsQL from the PEDFIC1 clinical trial were not used as there was a lack of consistency in the results. Trial participant numbers were small, especially among those self-reporting, and the mapping analysis was applied to aggregate data rather than patient-level data.  Due to a lack of HRQOL data from the PEDFIC1 clinical trial, the company identified values from closely related diseases.  Study by Kamath et al was used given the trial participant numbers and the availability of a mapping algorithm (54).  Published literature using the PedsQL used to model two health states. (54).  Company used Khan (2014) to map both the participant-reported and parent proxy-reported PedsQL score to the EQ-5D-3L (36)  A disutility associated with short stature is applied to ‘loss of response’ states from a HRQoL study in children with chronic kidney disease. | **ERG:** Issues with face validity of utility values.  **ERG:** The company’s mapping study would have been more appropriate than applying simplifying assumptions. The ERG also believes that these assumptions would overestimate QALYs associated with achieving response.  **Committee:** The ERG used the utilities from the company’s trial in its base case. The committee agreed the company’s utilities were likely high and preferred the utility values obtained directly from the clinical trial. |
| **ID 927** | Adults with EPP | EPP-QoL instrument used  The company estimated the cost per additional disability-adjusted life year. | **ERG:** Had concerns regarding the use of the EPP-QoL.  **ERG:** Used DLQI in the exploratory analysis as it has face validity |
| **ID 856** | Adults with severe alpha 1-proteinase inhibitor who have progressive lung disease | Data from published literature were used to obtain utility values (55).  HRQoL was assumed to be driven by forced expiratory volume, as opposed to lung density decline. | **ERG:** Expressed concerns regarding the generalisability of the published literature used to inform utility values (55).  **ERG:** Expressed concerns over the age-related utility decrements.  **ERG:** Criticised the company for not attempting to model the impact of lung density decline on people with the disease. |
| **ID 800** | People with alpha-mannosidosis aged six years and over | EQ-5D-5L questionnaire completed by clinicians as a proxy | **ERG:** Raised concerns that the utilities lacked face validity.  To address these concerns the company commissioned a survey to provide additional data on the utility within each health state |
| **ID 737** | People with lysosomal acid lipase deficiency | Utilities were identified from published literature (56). | **ERG**: Proposed two adjustments to the application of health utility values: a cap on the health-utility at the level of the age-matched general UK population and the use of health state utilities from an alternative source (57). |
| **ID 1590** | Inoperable plexiform neurofibromas associated with type one neurofibromatosis in children aged 3 years and over | Vignette Study | As described in Table S2 |
| **ID 1666** | Metachromatic leukodystrophy: in children with late infantile or early juvenile forms and in children with the early juvenile form with early clinical manifestations. | Vignette Study | As described in Table S2 |

*ADA-SCID, Adenosine Deaminase Deficiency–Severe Combined Immunodeficiency; AE, Adverse Events; AHP, Acute Hepatic Porphyria; aHUS, atypical Haemolytic Uraemic Syndrome; BOI, Burden of Illness; DCE, Discrete Choice Experiment; DLQI, Dermatology Life Quality Index; DMD, Duchenne Muscular Dystrophy; EPP, Erythropoietic Protoporphyria; EPP-QoL, EPP-Specific QoL; EQ-5D, EuroQoL-5 Dimension tool; ERG, Evidence Review Group; ERT: Enzyme Replacement Therapy, GD-DS3, Gaucher Disease Type one Severity Scoring System, hATTR, Hereditary Transthyretin Amyloidosis; HRQoL, Health Related Quality of Life;* *HST, Highly Specialised Technology; HUI, Health Utilities Index; ICER, Institute for Clinical & Economic Review; IVIG, Intravenous Immunoglobin; MAA, Managed Access Agreement; PedsQL, Paediatric Quality of Life Intervention; PFIC, Progressive Familial Intrahepatic Cholestasis; QALY, Quality-adjusted life years; RRMS, Relapsing-remitting Multiple Sclerosis;* *SF-36, Short Form Health Survey; SLR, Systematic Literature Review; SMA, Spinal Muscular Atrophy; THAOS, Transthyretin Amyloidosis Outcome Survey*

Table S2: Summary of HSTs using vignette studies

| **HST** | **Population** | **Vignette Description** | **ERG/Committee Comments** |
| --- | --- | --- | --- |
| **HST 5** | Adults with type 1 Gaucher disease | SF-6D used to inform health state utilities  Vignette used to inform disutility associated with infusion administration only (over oral administration) –  **Health state development:** Authors developed five different health state descriptions that were validated by a clinician and piloted on six members of the general public  **Health state valuation:** EQ-5D values elicited using time-trade off and 100 people from the general population | **ERG:** Company criticised for providing results of study only (no methods)  **ERG:** Wording of questions posed to participants to elicit utility valuations may have captured aspects unrelated to the mode of administration  **ERG:** Unclear whether benefits were due to improved health or administration method |
| **HST 6** | People with paediatric-onset hypophosphatasia | **Health state development:** Series  of detailed case studies were used by clinical experts to estimate utilities  **Health state valuation:** Derived from nine hypophosphatasia experts using patient vignettes  Valuation based on EQ-5D-5L | **ERG:** Utility values should have been obtained from people with the disease rather than from clinical experts  **ERG:** The values used were reasonable and had face validity  **ERG:** The company’s vignettes assumed a strong correlation among all dimensions of health which may have led to an underestimation of the true variation in health-related quality of life within each health state  **Committee:** The mean utility values used in the company's model were reasonable estimates for the 6MWT health states |
| **HST 8** | Children and young people with X-linked hypophosphatemia | **Health state development:** Four vignettes are described per age group (1 to 4 years, 5 to 12 years and adolescents & adults (13+))  A series of detailed case studies were developed which  included some clinical information as well as general information about functioning and symptoms  **Health state valuation:** six UK X-linked hypophosphatemia clinical experts  Valuation based on EQ-5D-5L (mapped to 3L)  Submitted new vignette study in an evaluation consultation document whereby experts valued the quality of life of people with the disease aged 18 years, 40 years and 60 years (using EQ-5D-5L) to model long-term disease progression | **ERG:** Each health state was defined in such a way that there appears to be a perfect association between the RSS score and other clinical descriptors of the health state (there is no possibility of improvement in one attribute with no change or even worsening of another) – not realistic  **ERG:** It is a limitation that utility values were obtained from clinical experts and not directly from people with the disease (or parents of children)  **Committee:** Preferred the additional evidence in the evaluation consultation document |
| **HST 11** | People with inherited retinal dystrophies caused by RPE65 gene mutations | **Health state development:** Descriptions developed with input from an advisory board, people with the disease & carers and clinician interviews; five vignettes described different levels of visual function in RPE65-mediated IRD, corresponding to each of the health states in the model  **Health state valuation:** Six clinicians were interviewed to provide a proxy valuation of each vignette using the HUI3 and EQ-5D-5L questionnaires; the HUI3 was scored in line with developer instructions; the EQ-5D-5L  was scored using the van Hout el al algorithm  The HUI3 was preferred for the base case because the EQ-5D (3-level version) had previously been found to have poor convergent validity when used in visual disorders | **ERG & Committee:** Lack of patient-report values by people with the disease treated with the intervention was a key limitation  **ERG:** Following methodological issues with proxy elicitation: a limited number of respondents, clinicians may focus on issues related to vision loss only, completing ‘best health state’ first may lead to potential capping of utilities  **ERG:** Utility values did not have face validity – did not match values described by clinical advisors or previous submissions and a negative utility is unlikely  **Committee:** Disappointed that no direct measure of health-related quality of life had been used in the clinical trials |
| **HST 12** | People with a confirmed diagnosis of neuronal ceroid lipofuscinosis type 2 | **Health state development:** Separate vignettes were produced for each health state and treatment arm in the model (one describing a person with the disease at a given  health state being treated with cerliponase alfa and one describing an equivalent person being treated with standard of care)  **Health state valuation:**  Vignettes sent to eight clinical experts who were asked to complete the EQ-5D-5L questionnaire (mapped to EQ-5D-3L)  Utility values collected in company study (PedsQL) were not used in the model due to the small sample size and because utility values could not be obtained for all health states | **ERG:** Expressed concern that the vignettes imply significant additional benefits of the intervention, over and above the effects of disease progression  **ERG:** Concerned that the utility values in less severe health states are too high – exceed the adult general population and age-related decline not modelled  **ERG:** Concern regarding differences in utility values between EQ-5D-5L and mapped EQ-5D-3L (EQ-5D-5L may be more reflective of reality)  **ERG:** The vignettes appear to underestimate utility when compared to the PedsQL. Although the PedsQL is bound at zero, it aligns better to the mapped EQ-5D-3L. Concluded that the vignettes values were most suitable  **Committee:** Requested further data collection within MAA due to uncertainty associated with health-related quality of life (specific patient-reported outcomes measures requested are confidential) |
| **HST 13** | Adults with confirmed familial chylomicronaemia syndrome | EQ-5D data collected in company study was not used in the model due to implausible values (higher than UK average)  **Health state development:** Developed with clinical and patient experts  **Health state valuation:** Seven clinical experts assessed vignettes | **ERG:** Utility for a person within a health state should not depend on whether the person is on treatment as assumed in the company’s base case  **ERG:** Prefers utilities more aligned to the vignette results (not dependent on whether the person is on treatment or not)  **Committee:** Recognised intermittent symptoms of FCS might explain why a one-off questionnaire might not fully capture the treatment effect on quality of life, but did not believe the clinical trial results showed that volanesorsen did not affect quality of life |
| **HST 15** | Children with SMA type 1 | Base case utility values informed from a US ICER assessment of SMA therapies. Utility values from three alternative sources included as scenarios only: PedsQL mapped to EQ-5D-y, clinician assessed case vignette study EQ-5D-y, parent proxy EQ-5D-3L  **Vignette health state development:** Four health states developed reflecting model health states  **Vignette health state valuation:** Face to face interviews with 100 adults: visual analogue scale (anchored at -100 and +100) followed by time trade off for two scenarios: parent vignettes and adult vignettes  Company noted that vignettes were lengthy, making them hard to read, contributing to severe health states rated similarly. Adult vignettes were also lower than parent proxy vignettes. Therefore, NOT used in the base case  Alternative elicitation: participants were asked to complete visual analogue scale and time trade-off imagining that they were a parent of a child with SMA, and an adult with SMA. ‘Parent’ vignettes considered most appropriate study | **ERG:** Acknowledged health-related quality of life estimation is problematic for this condition and considered it was appropriate to provide a wide range of plausible scenarios  **Committee:** Understood that obtaining robust health-utility values was challenging and considered that the uncertainty around the health-state utilities used in the model had a major effect on estimates of cost-effectiveness  **Committee:** Considered that the additional on-treatment utility was appropriate in the analysis in this situation but only because study outcomes showed benefits beyond that captured in the health states |
| **HST 17** | People with progressive familial intrahepatic cholestasis | Utilities informed by literature in base case  Company is undertaking a valuation study following advice received from NICE/ERG during the decision problem meeting  **Vignette health state development:** Qualitative interviews were conducted with clinicians to obtain feedback on different health state vignettes  **Vignette health state valuation:** Vignettes will then be used in time trade-off interviews with general public | **ERG:** Expressed a preference for mapping the PedsQL data collected within the clinical trial within the base case  **Committee:** Agreed with company’s use of utilities informed by literature in the base case |
| **ID1666** | Metachromatic leukodystrophy: children with late infantile/early juvenile and early juvenile with early clinical manifestations. | **Vignette health state development:** Vignettes were informed by clinical experts and patient groups and validated by clinicians at an advisory board  **Vignette health state valuation:** Utility values elicited from members of the public using the time trade-off method | **ERG:** Considered the approach adopted by the company to be inconsistent with the NICE reference case and that the utility values generated were unfit for decision making (the resulting value set captured only public preferences with no explicit consideration of the patients themselves)  **ERG:** Noted issues with the vignettes – responses tended towards the best and worst possible ratings |
| **ID1590** | Inoperable plexiform neurofibromas associated with type 1 neurofibromatosis in children aged 3 years and over | **Vignette health state development:** Vignette descriptions based on a series of interviews with patients, parents/carers and clinical experts  Qualitative interviews conducted with adult patients and parents/carers of paediatric patients to validate health states  **Vignette health state valuation:** Interviews with general public used to estimate utility values using the time trade-off method (used in base case) and visual analogue scale | **ERG:** Critical that utility values were not informed using patient-reported data  **ERG:** Lengthy health state descriptions could have led to heuristic shortcuts and use of bolding may have focussed participants on the more positive elements of the treated state and the more negative elements of the untreated state |

*6MWT, 6-minute walk test;* *EQ-5D, EuroQoL-5 Dimension tool; ERG, Evidence Review Group; FCS, familial chylomicronaemia; HST, Highly Specialised Technology; HUI3, health utilities indix mark 3; ICER, Institute for Clinical & Economic Review;* *PedsQL, Paediatric Quality of Life Intervention; RPE65-mediated IRD, RPE65-mediated inherited retinal dystrophy; RSS, Ricketts Severity Score, SF-36, Short Form Health Survey;* *SMA, Spinal Muscular Atrophy*

Table S3: Summary description of the utility sources for HRQoL for caregivers

| **HST** | **Population** | **Description of Caregiver HRQoL** | **ERG/Committee Comments** |
| --- | --- | --- | --- |
| **HST 2** | People with Mucopolysaccharidosis type Iva | BOI study matched to corresponding EDSS to give a proxy value (58).  **Included as scenario analysis only.** | **ERG**: No major concerns with the approach used. |
| **HST 3** | People 50-years and older with DMD | Disutilities obtained from Landfelt *et al* (47). The company identified this as a weakness but suggested reports this paper may have underestimated the disutility of certain health states for caregivers.  **After consultation:** Disutilities applied for three caregivers rather than one caregiver  **Included as scenario analysis only.** | **ERG:** The ERG reviewed the company’s model after consultation and made some amendments including applying caregiver disutilities for two caregivers rather than three, as the ERG was unclear as to why the same disutility for one primary caregiver should be applied to all caregivers.  **Committee:** The committee noted that the proposed managed access agreement would capture caregiver quality-of-life data and considered it imperative that its future review of guidance includes these data (given the large associated effect on the cost consequence model). |
| **HST 9** | People with hATTR | Disutilities applied from published literature: Gani *et al* developed an algorithm which calculated carer disutility. This attributed a rising disutility for carers as severity worsened (59).  Proxy: Carer disutility by EDSS severity score in MS.  Two caregivers assumed in model (Company cited HST 3 as justification for applying disutility to more than one caregiver).  **Included in base case analysis.** | **ERG**: Requested clarifications as to why the disutilities were applied to multiple caregivers as the company had also included homecare in the costs within the model.  **ERG:** Remains unclear whether all people with the disease would realistically have two caregivers. People in stage one would need minimal care and as people with hATTR spend most of their time in stage one and stage two health states assuming two caregivers throughout the model is inappropriate.  **Company response:** Provided two further analyses varying the number of caregivers between one and three caregivers. (One caregiver in stages one and two and two caregivers in stage three)  **Committee:** Accepted the company’s revised approach applying one caregiver for stages one and two and two caregivers for stage three of the model.  Assumptions around the number of caregivers had a modest impact on the ICER, ranging from £341,306 for three caregivers to £402,936 for one caregiver. |
| **HST 10** | People with hereditary transthyretin amyloidosis | Proxy caregiver disutility  Estimated disutilities from the tafamidis AGNSS report which was in turn based on an Alzheimer’s disease model previously reviewed by NICE (60).  **Included in base case analysis.** | **Committee:** Considered utilities modelled to be highly uncertain.  **Company response:** The company included one full time caregiver for stages one and two and two full time caregivers for stage three. The committee was satisfied with this (this method was accepted also in HST 9). |
| **HST 11** | People with inherited retinal dystrophies caused by RPE65 gene mutations | Disutilities obtained from published literature:  Kuhthau *et al*. 2010 (61)  Different disutilities applied to carers of adult and paediatric people with the disease.  **Included as scenario analysis only.** | **ERG:** Uncertainty associated with the approach, but broadly agreed with the approach taken by the company.  **ERG:** School-aged children would typically have more than one caregiver, disutility was multiplied by 1.78 (the mean number of parents in a household). |
| **HST 12** | People with a confirmed diagnosis of neuronal ceroid lipofuscinosis type two | Sibling disutility was applied across all but the first two health states in line with clinical guidance.  Average number of caregivers in the model varied by different health states (elicited from a Delphi panel).  **Included in base case analysis.** | **ERG:** Criticised the disutility being applied over a lifetime  **ERG:** The ERG considered the inclusion of caregiver and sibling disutilities to be appropriate given the evidence provided regarding the substantial impact of the condition on family life. Also noted removing the caregiver/ sibling disutility had minimal impact on the ICER. |
| **HST 13** | Adults with confirmed familial chylomicronaemia syndrome | Proxy disutility obtained from previous HST submission:  Metreleptin HST (26)  **After consultation:** the company presented an alternative utility value. This decreased the decrement from 0.1 to 0.04.  **Included in base case analysis.** | The committee and the ERG were concerned about the source of the proxy value disutility. Both the committee and the ERG were also concerned around the value itself.  **Committee:** the committee asked the company to explore alternative values.  E**RG:** The ERG remained concerned that the new utility decrement might still be an overestimation |
| **HST 14** | People with generalised or partial lipodystrophy | **Base Case:** Described qualitatively  **Scenario Analysis:**  Disutility obtained from DMD submission (HST 3).  Assumed two caregivers (rounded median number of carers: 1.67 caregivers).  **Included as scenario analysis only.** | **ERG:** Used 1.67 carers in its base-case analysis.  **Committee:** Agreed with the ERG’s approach and stated that not all people with lipodystrophy will have two carers. |
| **HST 15** | Children with SMA type one | Proxy disutilities used (based upon spina bifida) (62).  Disutilities varied per different health states.  Sensitivity analysis showed the disutilities used had a small impact on the ICER.  **Included as scenario analysis only.** | **ERG:** Clinical expert opinion reported SMA has a much higher burden than spina bifida because spina bifida is not associated with breathing or feeding difficulties.  **ERG:** Identified a Spanish study which assessed carer disutilities on SMA carers. The ERG’s alternative disutility was substantially larger than the company’s and may lack face validity.  **Committee:** Acknowledged onasemnogene would likely reduce carer burden in the short term. Also acknowledged uncertainties relating to future carer burden.  **Committee:** Concluded that accounting for carer HRQoL was important in appraising therapies for SMA but that their inclusion in economic modelling was complex |
| **HST 16** | Adults and young people aged 12 years or older with recurrent severe attacks of AHP | No studies were identified which reported caregiver disutility for AHP.  Disutilities obtained from published study which assessed HRQoL impact on carers for MS (63).  Assumed AHP health states to be equal to MS health states to estimate caregiver decrements  Data reported in the Acaster et al study (63). (Increasing caregiver disutility with increasing disease severity) Therefore, the caregiver disutility differed for each health state (company referenced this method being accepted in HST 2).  **Included as scenario analysis only.**  Alternative assumptions for disutilities were explored in scenario analysis  Two assumptions explored:   1. Caregiver disutility when the person was in the severe health state was equal to the recurrent health state 2. Caregiver disutility for asymptomatic and symptomatic health states was set to zero | **ERG:** The ERG was unclear as to whether the carer disutilities included in the model were appropriate: clinical advisors were uncertain as to whether people would need carers in all health states  **ERG:** Asked the company to clarify how disutilities were assumed to be equivalent to MS health states.  **ERG**: Overall considered there to be similarities between AHP and MS concerning the need for carers. |
| **HST 17** | People with progressive familial intrahepatic cholestasis | Proxy.  TA for SMA (NICE TA 588 and TA 534 (40))  1.78 carers per person with the disease  **Included in base case analysis.** | **ERG:** considered it an area of uncertainty to include caregiver disutilities in the analysis  **Committee:** Concluded that carer disutilities should be included in the modelling but that the extent of any carer disutility was uncertain |
| **ID 856** | Adults with severe alpha 1-proteinase inhibitor who have progressive lung disease | 5% reduction in related HRQoL was applied to people with FEV1 > 50% and 10% reduction was applied to all other health states.  **Considered in scenario analysis only.** | **ERG:** No direct comment. |
| **ID 800** | People with alpha-mannosidosis aged six years and over | **Key opinion leaders:**  Interviews.  EDSS. Gani *et al* (59).  Gani shows that carer disutility increases linearly as the disease becomes more severe.  One caregiver assumed.  **Included in base case analysis.** | **ERG**: Performed exploratory analysis excluding the caregiver disutilities |
| **ID 1590** | Inoperable plexiform neurofibromas associated with type one neurofibromatosis in children aged three years and over | Disutilities in the base case were assumption based.  Alternative assumptions were explored in scenario analysis using disutilities from other submissions.  **Number of caregivers:** 1.4  Average UK household 2.4 – patient = 1.4 caregivers.  **Included in base case analysis.** | **ERG:** Disutility was larger than disutilities applied in other appraisals  **ERG:** Did not accept the assumption that everyone in the household except the person with the disease would be a caregiver. The justification that ‘most other appraisals’ included one carer was considered appropriate. |
| **ID 1666** | Metachromatic leukodystrophy: in children with late infantile or early juvenile forms and in children with the early juvenile form with early clinical manifestations. | The EQ-5D-5L was completed by 21 carers.  Caregiver disutilities applied to most severe health states only (2 caregivers assumed).  **Included in base case analysis.** | **ERG:** Was satisfied with the application of caregiver decrements but considered it appropriate to apply disutilities from an earlier health state. |

*AGNSS, Advisory Group for National Specialised Services; AHP, Acute Hepatic Porphyria; BOI, Burden of Illness; DMD, Duchenne Muscular Dystrophy; EDSS, Extended Disability Status Scale; EQ-5D, EuroQoL-5 Dimension tool; ERG, Evidence Review Group; FEV1,* *forced expiratory volume in 1 second; hATTR, hereditary Transthyretin Amyloidosis; HRQoL, Health Related Quality of Life; HST, Highly Specialised Technology; ICER, Incremental Cost-Effectiveness Ratio; MS, Multiple Sclerosis; NICE, National Institute for Health and Care Excellence;* *SMA, Spinal Muscular Atrophy; TA, technology appraisal*
