# Supplementary material for: Uncertainties in evaluating the health-related quality of life and disease burden of people with rare diseases and their caregivers in NICE HST submissions
Source: Orphanet J Rare Dis. 2024 Oct 22;19:391. doi: 10.1186/s13023-024-03382-9 (PMC11494764; doi:10.1186/s13023-024-03382-9)
Supplement: Supplementary file 1 — Supplementary Material 1 [file 13023_2024_3382_MOESM1_ESM.docx]

18 March 2024

Francis Palau

Editor-in-Chief

Orphanet Journal of Rare Diseases

Dear Editor-in-Chief,

**RE:	Uncertainties in Evaluating the Health-Related Quality of Life and Disease Burden of People with Rare Diseases and their Caregivers in NICE HST Submissions**

We are pleased to submit this article on the uncertainties in evaluating health-related quality of life (HRQoL) and the disease burden of people with rare diseases and their caregivers in NICE Highly Specialised Technology submissions (HST) in The Orphanet Journal of Rare Diseases. The review leads on from the incentives for designated orphan products introduced by the European Union in 2000, and later the increased cost-effectiveness threshold proposed for NICE HST programmes. The review discusses the following challenges associated with treatments for rare diseases: demonstrating value for money, capturing the full health-related quality of life impact, mapping disease-specific measures to EQ-5D and estimating the disease burden of rare diseases through productivity costs. Our study reviews previous NICE HST appraisals and identifies commonly used methods to overcome the uncertainties in HRQoL and disease burden for people with rare diseases and their caregivers.

Our research into previous HST appraisals and their qualitative description of the impact of the disease burden on patient and caregiver HRQOL highlighted considerable uncertainty associated with the quantification of these aspects, specifically the uncertainty in methods used to obtain utility values. Our research suggests that EQ-5D remained the most well-received source of utilities, however, the evidence required to demonstrate the inappropriateness of using EQ-5D to capture the HRQoL impact of a rare disease is challenging for companies.

By submitting this review article for publication in The Orphanet Journal of Rare Diseases, we hope to communicate the need for new methodologies to reflect the impact of rare diseases on people with diseases and their caregivers, including a more flexible approach when EQ-5D is not appropriate and clear recommendations on the estimation of utilities across different rare diseases. In addition to this, we hope to highlight new ways in which to evaluate paediatric HRQoL, further research and methodological advances to quantify bereavement and identify ways to quantify disease burden.

We confirm that all authors have approved the manuscript for submission and that the content of the manuscript has not been published or submitted for publication elsewhere.

Thank you for considering our manuscript for publication in The Orphanet Journal of Rare Diseases.

Yours sincerely,

**Kinga Malottki**
